# Epidemiology of dermatophytosis in northeastern Iran; A subtropical region

**DOI:** 10.18502/cmm.5.2.1156

**Published:** 2019-06

**Authors:** Maryam Ebrahimi, Hossein Zarrinfar, Ali Naseri, Mohammad Javad Najafzadeh, Abdolmajid Fata, Mahmoud Parian, Imaneh Khorsand, Monika Novak Babič

**Affiliations:** 1 Department of Biology, Damghan Branch, Islamic Azad University of Damghan, Damghan, Iran; 2 Allergy Research Center, Mashhad University of Medical Sciences, Mashhad, Iran; 3 Department of Parasitology and Mycology, School of Medicine, Mashhad University of Medical Sciences, Mashhad, Iran; 4 Cutaneous Leishmaniasis Research Center, School of Medicine, Mashhad University of Medical Sciences, Mashhad, Iran; 5 Department of Biology, Biotechnical Faculty, University of Ljubljana, Ljubljana, Slovenia

**Keywords:** Dermatophyte, Dermatophytosis, PCR-RFLP, Subtropical, Iran

## Abstract

**Background and Purpose:**

Dermatophytes as the causative agents of dermatophytosis (ringworm) are widely spread around the world. Accurate identification of dermatophytes in one area can be particularly important for epidemiological studies. Regarding this, the aim of the present study was to describe the species spectrum of dermatophytes, isolated from patients in Mashhad city, Iran, using the molecular-based method.

**Materials and Methods:**

This study was conducted on 79 dermatophyte isolates obtained from the human skin, hair, and nail specimens. Species identification was performed by the polymerase chain reaction-restriction fragment length polymorphism analysis of ribosomal DNA **internal transcribed spacer** regions using *Mva*I restriction enzyme.

**Results:**

The identified species included *Trichophyton mentagrophytes*/*T. interdigitale *species complex (n=37, 46.8%), *Epidermophyton floccosum *(n=12, 15.2%), *T. rubrum* (n=8, 10.1%), *Microsporum canis *(n=8, 10.1%), *T. violaceum *(n=5, 6.3%), *T. tonsurans *(n=4, 5.1%), *Nannizzia gypsea * (n=3, 3.8%), *T. benhamiae * (n=1, 1.3%)*,* and *T. verrucosum * (n=1, 1.3%). The clinical forms of infection were tinea corporis (n=26, 32.8%), tinea cruris (n=22, 27.8%), tinea capitis (n=10, 12.6%), tinea unguium (n=7, 9%), tinea manuum (n=6, 8%), tinea pedis (n=5, 6.3%), and tinea faciei (n=3, 3.5%).

**Conclusion:**

As the findings indicated, *T. mentagrophytes*/*T. interdigitale *species complex had the highest prevalence, and *T. benhamiae* appeared to be a new emerging agent of dermatophytosis in Mashhad, northeastern Iran.

## Introduction

Dermatophytes are a group of keratinophilic molds with a global distribution that can invade the keratineous materials in the outer layer of the skin and its appendage structures, such as the hair, nails, hooves, feathers, and claws, in humans and animals. These molds cause a spectrum of infections known as dermatophytosis (i.e., ringworm or tinea) [1]. Based on the most recent introduced taxonomy, this group consists of more than 50 species distributed in the genera of *Trichophyton*, *Microsporum*, *Epidermophyton*, *Nannizzia*, *Arthroderma*, *Lophophyton*, *Paraphyton, *and* Guarromyces *[2]*. *

Over the past century, the distribution of dermatophytes isolated from clinical specimens has undergone a significant change. The spectrum of species varies significantly from country to country [3]. A number of factors are responsible for the distribution of dermatophytes, including high population density and social activities in rural and urban areas*, low living* *standards,* *and* the *growth* of *immigrant* populations [4]. **The **ecological changes, **migration, **international travel,** and socioeconomic alterations can evolve the epidemiological aspects [**5**].** Dermatophytes account for human and animal infections with diverse clinical manifestations and can be transmitted via various routes. 

Although the infection is not life-threatening, it can sometimes be serious, as in the case of deep dermatophytosis [6]. Identification and differentiation of dermatophyte species are important from an epidemiological point of view. The wide use of empirical antifungal agents in clinical practice has resulted in a varied pattern of antifungal susceptibility among particular dermatophyte species [7]. **Regarding this, the identification of the causative agents and potential sources of **dermatophytosis **is an issue of significant importance facilitating the accurate control and treatment of this infection [**8**].** **The spread of **dermatophyte species** in all parts of the world, especially the Middle East, has not been fully understood yet.**

**Currently, **the identification of dermatophytes in the majority of the medical mycology laboratories in Iran is mostly based on the macroscopic and microscopic *characteristics* of the isolated colonies, which render imprecise results that are not identical to the current taxonomy of dermatophytes [9, 10]. With his background in mind, the present study was conducted to characterize the mycological and clinical aspects of dermatophytosis in Mashhad, a subtropical region of northeastern Iran, using the molecular-based method.

## Materials and Methods

**This study was approved by the Ethics Committee of Mashhad University of Medical Sciences, Mashhad, Iran (Ethics Committee code: IR.MUMS.REC.1392.34). **This research was conducted on the skin, hair, and nail clinical specimens collected from the patients (suspected of dermatophytosis) referred to the medical mycology laboratories of Ghaem and Imam Reza University hospitals in Mashhad** during 2014-2015**. The samples were examined using 15**% potassium hydroxide **and cultured on Sabouraud dextrose agar with chloramphenicol and cycloheximide medium (Conda, Spain). The cultures were then incubated at 28-30°C for 21-28 days, which resulted in the achievement of 79 dermatophyte colonies **from the patients with dermatophytosis. **

**For DNA extraction, a small piece of fresh dermatophyte colony** *was* *placed* in a *1.5*-mL Eppendorf tube, containing glass beads (0.5 mm) and lysis buffer (200 mM Tris-HCl, pH of 7.5, 25 mM EDTA, 0.5% w/v SDS, and 250 mM NaCl), and then homogenized using a homogenizer (SpeedMill Plus, **Jena, Germany**) according to the manufacturer’s protocol. In the next stage, the genomic DNA was **purified by** the phenol-chloroform method **[**5**]. The internal transcribed spacers (ITS) 1 and 2 regions, and the 5.8S ribosomal DNA subunit were amplified using two universal fungal primers, namely ITS1 (5´-TCCGTAGGTGAACCTGCGG-3´) and ITS4 (5´-TCCTCCGCTTATTGATATGC-3´) [**11**, **12**]. The amplification of DNA was accomplished using polymerase chain reaction (PCR) as previously described by Rezaei-Matehkolaei *et al*.** **[**11**]. The PCR products were digested by *Mva*I FastDigest restriction enzyme (Fermentans Life Sciences, Lithuania) **at *37°*C for *10 min [*11*].*

*The restriction products were separated by electrophoresis in 2% agarose gels, and the size of DNA fragments was compared with those reported in the previous studies [* 11 *].* **To confirm the accuracy and efficacy of **PCR-*restriction fragment length* *polymorphism* *(RFLP)* results** in **dermatophyte** identification, 11 isolates were randomly subjected to ITS sequencing. The PCR products were cleaned **from primers, nucleotides, polymerases, and salts** by means of a QIAquick purification kit (Qiagen GmbH, Hilden, Germany), and then sequenced on an ABI PrismTM 3730 genetic analyzer (Applied Biosystems, Foster City, CA, USA) with the ITS1/ITS4 primers. The obtained sequences were identified at the species level by using the validated Online Dermatophyte Database of the Westerdijk Fungal Biodiversity Institute at Utrecht, the Netherlands (www.westerdijkinstitute.nl).**

## Results

The results of direct examination were positive for all of the 79 dermatophyte colonies collected from the patients with suspected dermatophytosis regarding the presence of septate *hyphae* and/or *arthroconidia*. The clinical specimens consisted of 62, 10, and 7 skin, hair, and nail samples, respectively. Among the patients with dermatophytosis, 66% of the cases were male (*n*=52). The patients were within the age range of 1-98 years with the highest frequency (21.5%) in the age group of 21-30 years (Table 1). The spectrum of clinical presentations included tinea corporis, tinea cruris, tinea capitis, tinea unguium, tinea manuum, tinea pedis, and tinea faciei. Table 2 presents the clinical presentations and their causative agents.

The electrophoresis of the PCR-RFLP products revealed different banding patterns that were confirmed to belong to nine various dermatophyte species after ITS sequencing. These dermatophyte species included *T. mentagrophytes*/*T. interdigitale *species complex, *E. floccosum*, *T. rubrum*, *M. canis*, *T. violaceum*, *T. tonsurans*, *N. gypsea*, *T. benhamiae,* and *T. verrucosum *(Table 2). *On the other hand, because* T. mentagrophytes and T. interdigitale *had the same ITS electrophoretic pattern after digestion with *MvaI*, they could not be differentiated by ITS RFLP. As a result, all of the isolates with such patterns were reported as* *T. mentagrophytes*/*T. interdigitale *species complex. *The results of the complete* ITS *region* sequences of the isolates were submitted to the GenBank under the accession numbers of MF850250/53 and MH790392/98. 

**Table 1:** Prevalence of different clinical forms of dermatophytosis among various age groups in Mashhad, Iran

	**1-10 years**	**11-20 years**	**21-30 years**	**31-40 years**	**41-50 years**	**51-60 years**	**> 61 years**	**Total (%)**	***P-value***
Tinea corporis	3	5	6	6	4	1	1	26 (32.8)	0.713
Tinea cruris	0	3	8	4	2	3	2	22 (27.8)	0.106
Tinea capitis	8	2	0	0	0	0	0	10 (12.6)	<0.001
Tinea unguium	0	0	1	2	1	2	1	7 (9)	0.171
Tinea manum	0	3	2	1	0	0	0	6 (8)	0.602
Tinea pedis	0	0	0	1	1	1	2	5 (6.3)	0.028
Tinea faciei	1	2	0	0	0	0	0	3 (3.5)	0.455

Except for tinea capitis and tinea pedis, the clinical forms of dermatophytosis have the same distribution across different age groups.

**Table 2:** Frequency of different clinical forms of dermatophytosis in association with causative agents in Mashhad, Iran

**Dermatophytes**	**Clinical forms**							**Total (%)**
	**Tinea corporis (%)**	**Tinea cruris (%)**	**Tinea capitis (%)**	**Tinea manuum (%)**	**Tinea unguium (%)**	**Tinea pedis (%)**	**Tinea faciei (%)**	
*E. floccosum *	2 (7.7)	8 (36)	0	0	2 (29)	1 (20)	0	12 (15.2)
*M. canis *	2 (7.7)	0	3 (30)	1 (16.7)	0	0	0	8 (10.1)
*N. gypsea *	0	0	1 (10)	1 (16.7)	0	0	0	3 (3.8)
*T. benhamiae *	0	0	0	1 (16.7)	0	0	0	1 (1.3)
*T. mentagrophytes*/ *T. interdigitale *	17 (65)	9 (41)	4 (40)	1 (16.7)	3 (42)	3 (60)	2 (67)	37 (46.8)
*T. rubrum *	3 (11.9)	2 (9.1)	0	2 (33.2)	2 (29)	0	1 (33)	8 (10.1)
*T. tonsurans *	0	2 (9.1)	1 (10)	0	0	0	0	4 (5.1)
*T. verrucosum *	0	1 (4.8)	0	0	0	0	0	1 (1.3)
*T. violaceum *	2 (7.7)	0	1 (10)	0	0	1 (20)	0	5 (6.3)

## Discussion

**Dermatophytes are an important group of the skin, hair, and nail pathogens that can cause some serious problems as a result of deficient sanitation. The **distribution of dermatophyte species varies across different geographical regions.** Regarding this, the **accurate identification of dermatophyte species in a particular region can clarify the epidemiological aspects. In the current study, the cutaneous specimens obtained from the patients with suspected dermatophytosis were examined in Mashhad.

Based on the evidence, the causative agents of dermatophytosis correspond to a group of nearly 7 different genera with more than 50 species, 11 cases of which are most commonly reported in humans [2]. However, *T. mentagrophytes*/*T. interdigitale *species complex and *T. rubrum* are together responsible for more than 80% of all cases of dermatophytosis around the world [13, 3]. Over the past two decades, tremendous changes have taken place in the classification, *taxonomy*, and nomination of dermatophytes [2]. However, in some parts of the world, the dermatophytes are still identified by the conventional phenotypic methods, which mostly present unreliable results [14]. 

**The identification of dermatophytes based on phenotypic techniques not only requires experienced technologists but also is often labor-intensive with prolonged turnaround time. Moreover, these methods cannot be used for the complete differentiation of species within the genus or subspecies. **In some recent investigations carried out in Iran, the identification of these fungi has been accomplished by the sequence-based methods [5, 8]. However, to the best of our knowledge, there are no data regarding the mycological aspects of dermatophytosis in Mashhad based on the DNA-based method [14]. Regarding this, the present study involved the characterization of the identity of dermatophytes causing dermatophytosis in Mashhad by means of this method. 

The distribution of dermatophyte species varies depending on the climate and geographical location. Moreover, it seems that overcrowding, human-*animal* *interaction patterns *of *children*, and poor economic conditions are the significant underlying factors for this infection [15]. For example, **in the United States, ***T. rubrum* was **reported as the major causative agent of dermatophytosis, while *T. violaceum *has been introduced as the dominant etiological agent in most of the African countries [**16**, **17**]. However, in many parts of the world, there has been a lot of changes in the spread of certain species. **

**For instance, **the incidence of dermatophytosis due to *M. canis* has strongly increased in Europe during recent years [3]. There is also a discrepancy between the results of a study conducted in Mashhad by Naseri *et al*. [14] and those of the current study about **the major causative agents of dermatophytosis.** **In the current study, the **prevalence of* T. mentagrophytes*/*T. interdigitale* was higher than that of other species, while in the study by Naseri *et al*., *E. floccosum* was the dominant dermatophyte. The *difference in the prevalence rate can be due to several factors, including changes in the living conditions and cultures,* increased prevalence of migration and travel, and use of new identification methods. 

**Dermatophytes can** **affect** **both genders and all age groups in different regions; nonetheless, based on the local and international scientific reports, dermato-phytosis occurs predominantly in males [** 5 **,** 18 **-20]. In the same vein, the results of the current study indicated a higher incidence of dermatophytosis among the male patients (66%).** **This could be due to the higher involvement of men in outdoor activities. In this regard, it seems that the individuals who deal with domestic animals and soil are more likely to be infected with these fungi [**21**].**

**One of the limitations of this study was the lack of comprehensive information on the occupational status of the patients; in addition, this study had a small sample size. The clinical forms of dermatophytosis vary across the studies conducted in Iran and around the world [** 8 **,** 22 **].** **Even the severity of clinical symptoms **can be different due to the *species* and *strain* of the *dermatophytes* causing the infection [23]. In the present study, the most common clinical form was tinea corporis, followed by tinea cruris, which** is in agreement with the previous reports conducted in Iran and other countries [**5**, **24**-**27**]. **

**Currently, tinea corporis is reported as the dominant clinical form of dermatophytosis in the Middle East [** 5 **,** 22 **]. This form is **often acquired by close person‐to‐person contact. Accordingly, some specific social relationships can exert a great influence on the distribution of this infection in this region. However, the lesions caused by geophilic and zoophilic dermatophytes can produce a more intense inflammatory response than those caused by anthropophilic species [28]. Unlike many worldwide reports introducing *T. rubrum* as the predominant cause of infection [3], *T. mentagrophytes*/*T. interdigitale* species complex were the dominant agents of dermatophytosis in Mashhad. This is in accordance with the results of the most recent DNA-based studies performed in Tehran, Ahvaz, Mazandaran province, and Isfahan in Iran [5, 8, 29, 30], as well as those of the other reports [22, 31]. 

*T.* *rubrum*, *T. violaceum*, *E. floccosum*, and *M. canis* were identified as the other agents of tinea corporis among dermatophyte isolates. This is contrary to the reports from Europe where most of the tinea corporis cases were due to *Microsporum* species, especially *M. canis* [13, 32]. In the past, *M. canis* was one of the most prevalent agents of scalp infection in Iran [34]; however, recently, this infection has been reported to be caused by species other than *M. canis* (e.g., *T. mentagrophytes* and *T. tonsurans*) [5, 22]. The growing trend of *keeping pets* (e.g., *dogs* and *cats*) at home can be one of the main causes of the increased incidence of *M. canis *in this area.

The second most common clinical form among the patients with dermatophytosis was tinea cruris (groin) or jock itch, which is in agreement with the results reported in other studies conducted in Iran [5, 22, 34]. Although according to some reports, the patients with tinea cruris often have concurrent dermatophyte infections of the feet, in the present study, those cases were not accounted [35]. Based on the evidence, the infection usually affects adult men [5, 36]; likewise, in this study, 80% of the patients were male. **The type of dermatophyte species causing the infection varies in different ***geographical regions* around the world**. While ***E. floccosum* and *T. rubrum* are reported as the **common dermatophytes **[37]**, in the current study, the most causative agents were ***T. mentagrophytes*/ *T. interdigitale *species complex and *E. floccosum***, respectively.** **This difference can be due to various factors, including the number of samples, geographical area, population density, and climate conditions. **

**In the current study, tinea pedis had the lowest frequency (7%) in comparison to the other clinical forms. However, Toukabri** ***et al.*** **(22.5%) and Vena** ***et al.*** **(20.4%) reported higher incidence rates for this clinical form [** 38 **,** 39 **]. The prevalence of the infection is expected to undergo a dramatic increase owing to the increasing urban population** and sports activities**. On the other hand, the relevant environmental factors, such as **pH and CO_2_ concentration,** may be effective in this regard [**40**]. In the current study, ***T. mentagrophytes*/*T. interdigitale *complex was the main causative agent of most of the clinical forms, probably due to the high prevalence of the fungus in this area. **On the contrary,** *T. rubrum* has been reported as the main species implicated in tinea pedis in previous global studies [3,41-42]. 

As our results indicated,* T. benhamiae* had the lowest prevalence, compared to the other species, and was reported in Mashhad for the first time. This fungus is rarely reported (or reported at a low frequency) in other studies conducted in Iran and the Middle East [5, 22]. One of the reasons can be the use of traditional identification methods and the subsequent misdiagnosis. This species was isolated from tinea manum in our research. In a study carried out by Rezaei-Matehkolaei [5] in Khuzestan, Iran, the species was also isolated from tinea manuum, tinea coporis, and tinea capitis. Given that the zoophilic species of *T. benhamiae *is a new strain that is recently derived from *T. mentagrophytes* complex, the lack of reports on this species can be justified. Our study was one of the first studies in Mashhad that used a molecular approach to identify the causes of dermatophytosis. The findings of the current research showed no significant difference in the distribution pattern of dermatophytosis and their causative agents between northeast Iran and the rest of the area.

## Conclusion

As the results of the present study indicated, *T. mentagrophytes*/*T. interdigitale *species complex and *E. floccosum* had the highest prevalence, compared to the rest of the dermatophytes. In addition, **tinea corporis** and tinea cruris were the most common clinical forms in the patients with dermatophytosis. Based on the findings, *T. benhamiae* appears to be a new emerging agent of dermatophytosis in the area under investigation. *However*, our findings should be confirmed by the implementation of further studies with a larger cohort.
